# The cell cycle checkpoint inhibitors in the treatment of leukemias

**DOI:** 10.1186/s13045-017-0443-x

**Published:** 2017-03-29

**Authors:** A. Ghelli Luserna di Rora’, I. Iacobucci, G. Martinelli

**Affiliations:** 1 Department of Hematology and Medical Sciences “L. and A. Seràgnoli”, Bologna University, Bologna, Italy; 2 Present: Department of Pathology, St. Jude Children’s Research Hospital, Memphis, TN USA

**Keywords:** DNA damage response, Checkpoint kinase inhibitor, Acute lymphoblastic leukemia, Acute myeloid leukemia, Chronic myeloid leukemia, Chronic lymphocytic leukemia

## Abstract

The inhibition of the DNA damage response (DDR) pathway in the treatment of cancers has recently reached an exciting stage with several cell cycle checkpoint inhibitors that are now being tested in several clinical trials in cancer patients. Although the great amount of pre-clinical and clinical data are from the solid tumor experience, only few studies have been done on leukemias using specific cell cycle checkpoint inhibitors. This review aims to summarize the most recent data found on the biological mechanisms of the response to DNA damages highlighting the role of the different elements of the DDR pathway in normal and cancer cells and focusing on the main genetic alteration or aberrant gene expression that has been found on acute and chronic leukemias. This review, for the first time, outlines the most important pre-clinical and clinical data available on the efficacy of cell cycle checkpoint inhibitors in single agent and in combination with different agents normally used for the treatment of acute and chronic leukemias.

## Background

### The DNA damage response (DDR) pathway

**Fig. 1:**
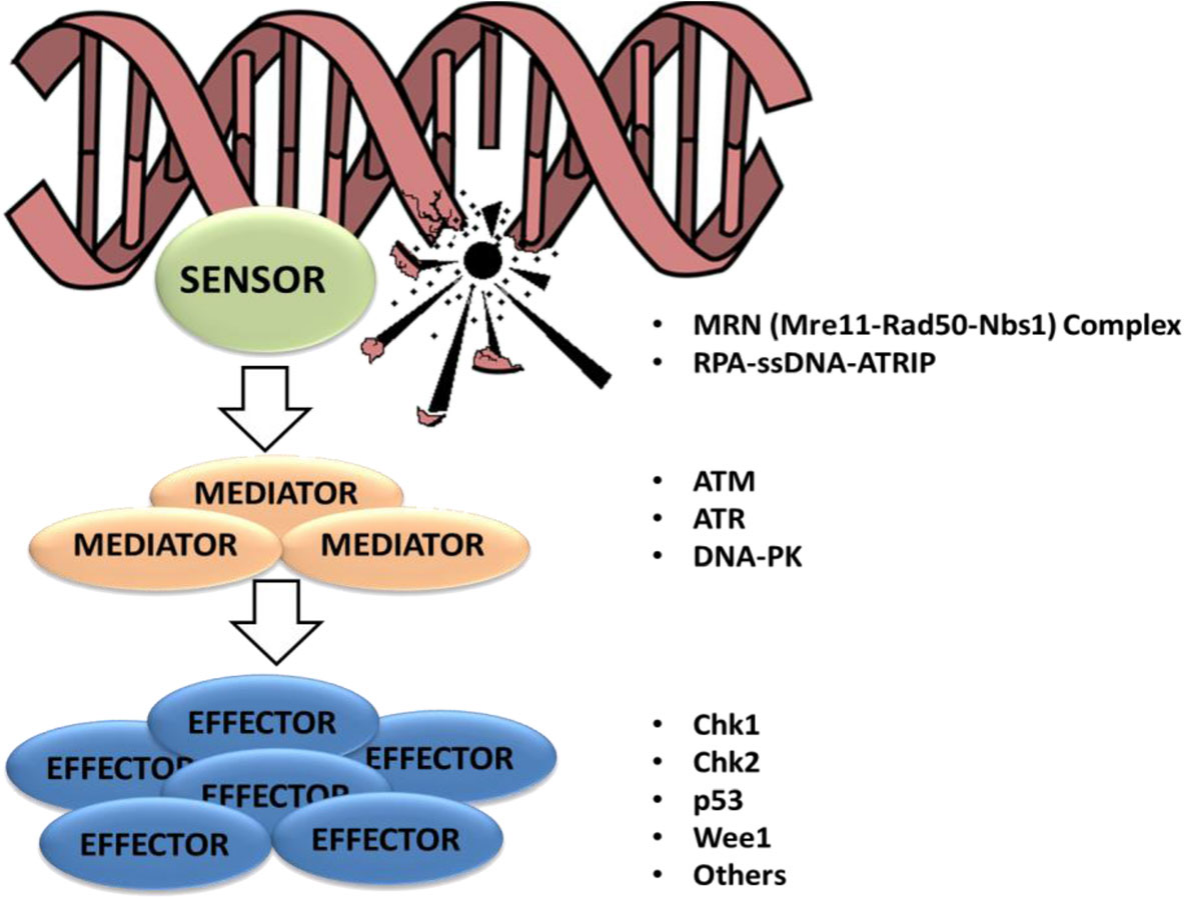
Schematic representation of the DNA damage response (DDR) pathway. DNA damages are sensed and repaired in multi-protein complexes. Signaling caused by this damage results in the activation of different mediators of the damage response and then results in cell cycle arrest and a choice between repair or progression to apoptosis

In the eukaryotic cells, the mechanism of response to DNA damages is generally termed DNA damage response (DDR) pathway (Fig. 1). The crucial function of the DDR pathway is to maintain genomic stability and to prevent tumor transformation. This pathway includes different regulators involved in the recognition of DNA damage (DNA damage sensors), in the recruitment of proteins on the site of DNA damages (DNA damage mediators) and in the response to DNA damages (DNA damage effectors) [1]. Three are the most important consequences of the DDR activation: (i) the regulation of the cell cycle, throughout the activation of different cell cycle checkpoints, (ii) the activation of the mechanisms of DNA repair and (iii) the induction of the apoptosis when the errors are too extended to be fixed.

#### DNA damage sensors and mediators

**Fig. 2:**
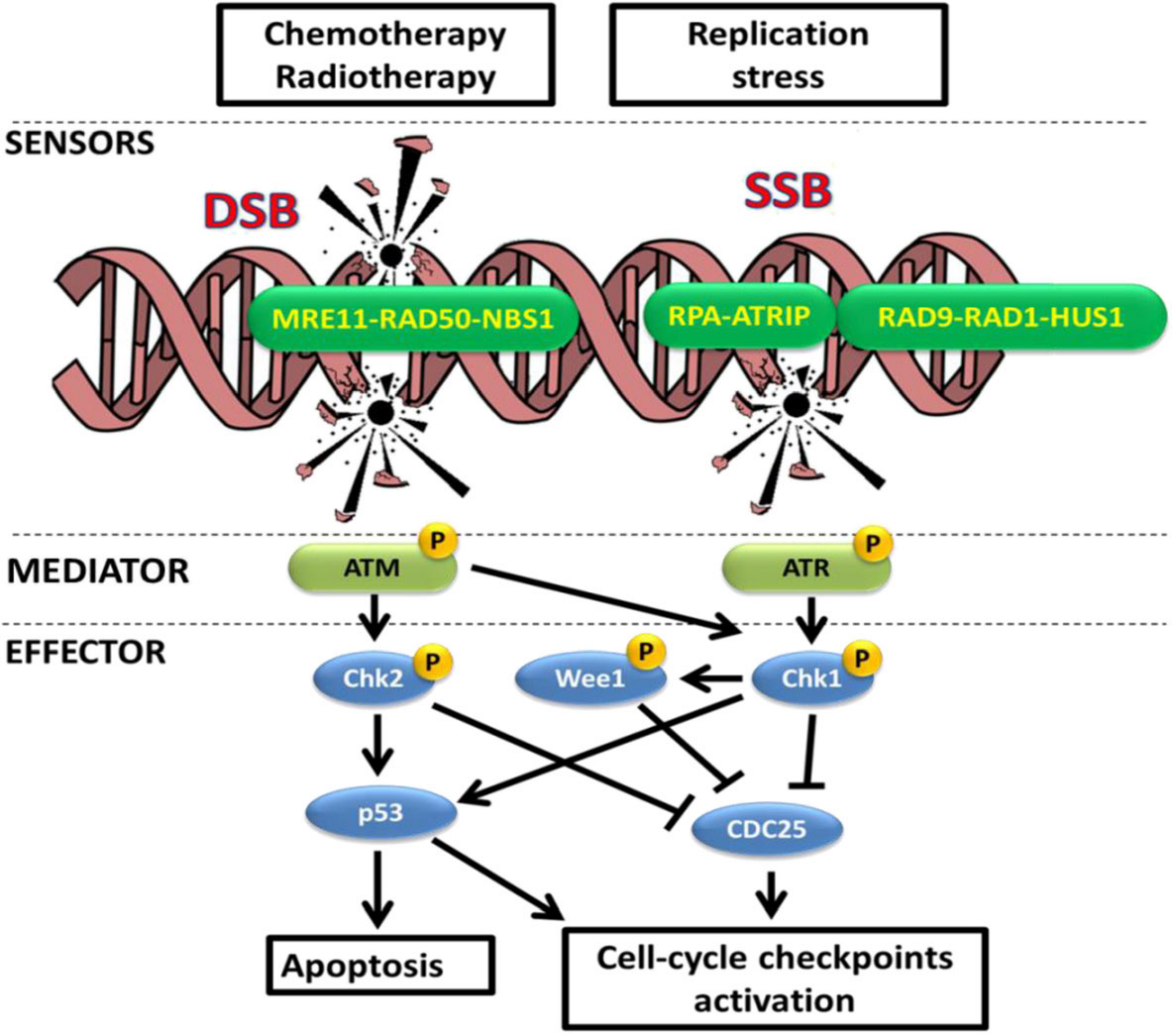
DNA damages sensor and mediators in the response to DSBs and SSBs. DNA damages trigger the recruitment of specific damage sensor protein complexes. On one hand, the MRN (MRE11–RAD50–NBS1) complex is required for the activation of ataxia-telangiectasia mutated (ATM) in response to double-strand breaks (DSBs). On the other hand, the ATM- and Rad3-related (ATR)-interacting protein (ATRIP) complex, formed by ATR-ATRIP-9-1-1 complex, is recruited to sites of single-strand breaks and activates ATR. The activation of ATM and ATR promotes respectively the activation of two different effectors, CHK2 and CHK1. Although currently, the activator of WEE1 is unknown, it is believed that CHK1 promotes WEE1 activation

Several deleterious attacks from extrinsic agents, such as ionizing radiations or genotoxic agents, as well as intrinsic sources, such as reactive oxygen species (ROS), can mine the DNA stability. In eukaryotic cells, appropriate intracellular levels of ROS play a crucial role in regulating several biologic processes. On the other hand, excessive production of ROS (due to primary oxidative metabolism in the mitochondria, metabolic processes, and inflammation) or inadequacy in a normal cell’s antioxidant defense system can cause oxidative stress and, eventually, DNA damages (ROS directly damages on DNA structure or base modification). Independently from the intrinsic or extrinsic sources, different types of DNA damage can be recognized, like adduction, strand torsions, or single breaks; however, the most deleterious are the double-strand breaks (DSBs) that arise when both the DNA strands are lesioned. Although DSBs are physiologically generated (i.e., immunoglobulin rearrangement or as a consequence of controlled oxidative metabolism), uncontrolled DSB generation is associated with genetic instability [2, 3]. Due to their high cytotoxicity, the generation of DSBs is the basis for conventional chemotherapy currently used in the treatment of different kinds of cancer [4]. Three kinases, members of the phosphoinositide three-kinase-related kinase (PIKK) family, the DNA-dependent protein kinase (DNA-PK), the ataxia-telangiectasia mutated (ATM), and the ATM and Rad3 related (ATR) have a relevant biological role in the initial phase of the DDR. In particular, both DNA-PK and ATM are involved in the response to DSBs while ATR is mostly involved in the response to DNA replication stress and in particular in the resolution of damages that involve only one strand of the DNA structure (single-strand breaks, SSBs) [5, 6]. In eukaryotic cells, the Mre11-Rad50-Nbs1 (MRN) complex is fundamental for the response to DSBs, for the localization of the sites of damages and for the activation of ATM itself. Indeed, it has been demonstrated that mutations, down-expression, degradation, or mislocalization of MRN components deeply affects ATM functionality [7–9]. MRE11 is a protein structurally composed by a Mn2+/Mg2+-dependent phosphoesterase domain and two DNA-binding domains [10]. The main function of MRE11 is to bind the DNA and, thanks to an exo- and endonuclease activity, to synapse the DNA ends [11]. RAD50 is a protein structurally homolog to a group of proteins involved in “the maintenance of the higher order structure of chromatin” called, SMC family proteins. The function of RAD50 is to maintain the DNA ends in close proximity thanks to an ATPase activity [12]. The third member of the MRN complex, NBS1, recruits different DNA repair and cell cycle checkpoint proteins (ATM itself) in the site of DNA damages [13]. In general, due to their central role in the early phase of the DSBs response, the members of the MRN complex are always present during the different phases of the cell cycle and are localized in a nuclear compartment known as promyelocytic leukemia (PML) bodies [14]. In presence of DSBs, MRN members rapidly, within seconds, delocalize from the PML bodies to the site of damages. Also, ATM is constitutively present in the nucleus of eukaryotic cells as an inactive dimer. In presence of DNA damages, ATM dissociates in active monomers and rapidly auto-phosphorylates multiple serine residues (Ser367, Ser1893, Ser1981, and Ser2996) to avoid the reconstitution of the inactive dimer [15, 16]. Further, post-transcriptional modifications like the acetylation of a lysine residue (Lys3016) and the phosphorylation of an additional threonine residue (Thr1885) complete the stabilization and the activation process [16]. ATM recruitment has been shown to require its binding to the C-terminus of NBS1, which is fundamental also for the kinase activity of ATM. When ATM is associated to the sites of damage, it rapidly phosphorylates the histone variant H2AX (ser139). This is a key event of both ATM and ATR transduction pathways and is necessary to amplify the signal of DNA damages and to facilitate the recruitment of other mediators of the DDR. Following the detection of a damage, ATM plays a central role in the activation of the G1/S cell cycle checkpoint, which prevents cells with damaged DNA from starting the S phase. This mechanism that will be better explained in the further section is primarily mediated through activation of the tumor suppressor protein p53 and of the checkpoint kinase 2 (CHK2). Single-strand DNA (ssDNA) is physiologically generated during DNA replication in all proliferating cells. Indeed, during the S phase, replication blocks are generated to allow the DNA polymerase to duplicate the two strands of DNA. The first event for the generation of the replication blocks is the activation of the replicative elicase; MCM (mini-chromosome maintenance) that ahead of the polymerase unwinds the double chain of DNA, generating ssDNA. Single strands are extremely fragile. Different insults can mine the stability of the replication forks, like the exposure to UV ray, causing the break of one strand and, consequently, generating SSBs. During DNA replication, the replicative blocks are keeping opened thanks to the activity of proteins termed replication protein A (RPA). These proteins bind the ssDNA and prevent the reconstitution of double helix. The first step of the response to SSB is the activation of ATR-interacting protein (ATRIP), the regulatory partner of ATR, directly binds RPA, thereby allowing the ATR–ATRIP complex to recognize the RPA-ssDNA at DNA damage sites or stressed replication forks [17]. The activation of ATR is strictly associated with the constitution of the ssDNA-RPA complex [18]. Then, the complex ATR-ATRIP-ssDNA-RPA stimulates the binding to the damage sites of second critical group of proteins, the RAD17/RFC2-5 clamp-loader complex. Consequently, the site of damage recruited the RAD9/HUS1/RAD1 (9–1–1) heterotrimer that in turn recruits topoisomerase II binding protein 1 (TopBP1) which activates ATR [19]. Recent studies have clarified on how TopBP1 engages and stimulates the ATR-ATRIP complex on RPA-ssDNA. Two independent studies showed that ATR is phosphorylated on a threonine residue (Thr1989) in the FAT domain after DNA damage, and this event is dependent to ATIP, RPA, and ATR itself activity [20, 21]. The interactions between TopBP1 and the ATR-ATRIP complex are believed to lead to conformational changes of the kinase that increases the activity of its kinase domain and/or its binding to substrates [22]. Once activated, ATM and ATR delay the cell cycle progression allowing the cells to resolve DNA damages before continuing the cell replication (Fig. 2).

#### DNA damage effectors

Different effectors are substrates of ATM and ATR kinases, and most of them are involved in cell cycle regulation (cell cycle checkpoint kinases) and in the mechanisms of DNA repair. Here, we will focus on the cell cycle regulation ATM/ATR-mediated. The most important substrates of ATR and ATM are the checkpoint kinase 1 (CHK1) and 2 (CHK2), respectively. CHK1 kinase is activated by ATR through the phosphorylation on serine 317 (ser317) and on serine 345 (ser345). Rapidly, CHK1 auto-phosphorylates on serine 296 stabilizing its structure and creating a binding site for the interaction with its direct substrates, the phosphatases CDC25 (CDC25A/B/C). The activation of CHK2 is enhanced by ATM through the phosphorylation on threonine 68 (thr68) and followed by several auto-phosphorylation events. CHK2 shares the substrate homology with CHK1 and inhibits CDC25A/B/C phosphatases in a similar way [23]. In eukaryotic cells, the cell cycle is finely regulated by the oscillation in the activity of different cyclin-dependent kinases, CDKs, which are positively regulated by proteins, called cyclins, and negatively regulated by CDK inhibitors (CDKI) and by a mechanisms of inhibitory phosphorylation [24, 25]. The transition from a phase of the cell cycle to another is regulated by different cell cycle checkpoints and in particular by the G1/S (transition through the G1 phase to the S phase), the intra-S and the G2/M checkpoints (transition to the G2 phase and entry in the mitosis). The activation of the G1/S checkpoint is mainly regulated through the activity of the tumor suppressor p53 which has been showed to be one of the direct substrate of ATM/ATR activation via the phosphorylation on serine 15 (ser15). Different sequential phosphorylations contribute to p53 stabilization and prevent the ubiquitination and consequently degradation enhanced by the negative regulator of p53, MDM2 [26]. The regulation of p53 in the G1/S checkpoint is also related to the activation of two direct substrates of both ATM and ATR, respectively, the checkpoint kinase 2 (CHK2) and the checkpoint kinase 1 (CHK1), that promote the activatory phosphorylation of p53 on serine 20 (ser20) [27, 28]. Once fully activated, p53 promotes the transcription of different genes involved in cell cycle regulation, like CDKN1A (cyclin-dependent kinase inhibitor 1A (P21, Cip1)), and induction of apoptosis, like BAX/PUMA/NOXA proteins [29]. The transition through the S phase is mainly regulated by a specific phosphatase, CDC25A [30]. This protein is necessary to remove the inhibitory phosphorylation on tyrosine 15 (tyr15) and threonine 14 (thr14) on cyclin-dependent kinase 2 (CDK2, CDC1). During normal replication, CDC25A activates CDK2 promoting the formation of the complex CDK2-cyclin E/cyclin A necessary for the entry into the S phase and for the DNA synthesis. In the presence of DNA damages, both CHK1 and CHK2 phosphorylate CDC25A on serine 136 (ser136) promoting its ubiquitination by SCF/TrCP ubiquitin ligase complex and following proteasomal degradation. The inhibition of CDC25A causes an S phase delay. Similar to the regulation of the S phase, also, the transition from the G2 to the M phase is strictly dependent on the activation of specific phosphates and in particular on the activation of both CDC25B and CDC25C. During the checkpoint activation, CDC25B is phosphorylated at serine 323 (ser323), by CHK1 and bound by 14-3-3 that blocks its catalytic activity [31, 32]. Both CHK1 and CHK2 negatively regulate CDC25C via phosphorylation of a serine residue (ser216); this event creates a site for the binding to 14-3-3 protein resulting in its cytoplasmic sequestration and G2/M checkpoint activation. The events that follow CDC25C sequestration are similar to those follow CDC25A degradation. The segregation of this phosphatase in the cytoplasm prevents its accumulation into the nucleus and consequently the inactivation of a protein complex crucial for the transition through the G2/M phase, the CDK1 (CDC2)-cyclin B complex. This complex is finely regulated not only by CHK1 or CHK2 but also by two proteins of the WEE1 family, WEE1, and MYT1. While both kinases can inhibit CDK1 through the phosphorylation on tyrosine 15 (Tyr15), MYT1 can also phosphorylate on threonine 14 (thr14), which has been shown to negatively regulate CDK1 as well. Thus, after the activation of the G2/M checkpoint, CHK1, CHK2, and WEE1 cooperate to negatively regulate CDK1 to prevent the formation of the complex with the cyclin B [33]. Although the regulation of WEE1 during normal cell cycle has been established [34], the mechanisms by which WEE1/MYT1 are activated in response to DNA damage in human is still not fully understood [35]. During normal cell division polo kinase 1 (PLK1) phosphorylates WEE1 promoting its degradation and, consequently, the beginning of mitosis. After DDR activation, both ATM and ATR promote the inhibitory phosphorylation of PLK1, leading to the nuclear accumulation of WEE1 [36].

### Cell cycle checkpoint-related proteins alteration in acute and chronic leukemias

Although in normal cells the cell cycle checkpoint kinases as well as other elements of the DDR pathway act as tumor suppressor and are crucial for the maintenance of genetic stability, in cancer, they have been found to protect tumor cells from different stresses and, consequently, to promote tumor progression [37]. Indeed, in normal cells, DNA errors are fixed by the repair mechanisms and if not, cell proliferation is arrested and cell death often ensues. The following section summarizes the main genetic alterations (mutations, copy number alteration, and gene expression alterations) that, although rare, have been reported in leukemias.

#### Mutations and copy number alterations in key cell cycle checkpoint genes in leukemias

The loss of function of ATM leads to the genetic disorder ataxia-telangiectasia (A-T), characterized by cerebellar degeneration, immunodeficiency, radiation sensitivity, chromosomal instability, and cancer pre-disposition [38, 39]. Mutations in ATM pre-dispose A-T patients to the development of lymphoid neoplasms, with a risk for leukemia approximately 70 times higher than the normal population [40]. Inactivating mutations and copy number alterations have been reported in both acute and chronic leukemia subtypes. In acute leukemia, Haidar and colleagues reported a high frequency of *ATM* deletions (10 out of 36; 28%), including 7 (19.4%) cases with loss of heterozygosity (LOH) and 3 (8.4%) cases with homozygous deletions in adult acute lymphoblastic leukemia (ALL) patients. Interestingly, in the ALL subgroup, the ATM protein deficiency (due to LOH or homozygous deletions) correlates with a favorable prognosis [41]. Copy number gains of *ATM* in 3 out of 191 (1.6%) adult patients with de novo acute myeloid leukemia (AML) have been reported by the Cancer Genome Atlas Research Network [42]. In chronic myeloid leukemia (CML), *ATM* was investigated as a potential candidate gene for the increased genetic instability following the evolution from chronic phase to blasts crisis (BC). Initial mutational analysis of 57 CML cases in BC highlighted no deleterious nucleotide changes in *ATM* and lack of correlation with BC progression [43]. However, the correlation between the loss of *ATM* and the acceleration of BC has been recently reported in CML mouse models [44]. LOH events involving the *ATM* locus and ATM protein deficiency occur in 14% and 34%, respectively, of patients with chronic lymphocytic leukemia (CLL) and have been found to correlate with aggressive disease and worse outcome [45]. Recent studies in large cohorts of CLL primary samples revealed a high frequency of missense/truncating mutation of *ATM* and deletion of *ATM* (associated with 11q22.3-23.2 deletion) [46–48]. *ATR* mutations, as well as copy number alterations, are rare in tumor cells due to the fundamental biological role of this kinase. Currently, no mutations affecting *ATR* have been annotated in acute and chronic leukemia patients, and only one case of single-nucleotide variant (SNV) out of 50 samples has been described in AML patients [49]. The downstream target of ATM, *CHK2*, has been found instead mutated in low rate in several kinds of cancer and in particular in hereditary cancers (*CHK2* 1100delC protein-truncating mutation confers a twofold increased risk of breast cancer) [50, 51]. In both acute (AML) and chronic (CLL) leukemias, only few studies reported mutations or copy number alterations of *CHK2* and with a very low percentage [52–54]. Similarly to *ATR*, no mutations have been reported in *CHK1* in acute and chronic leukemias.

#### Gene expression alteration of key cell cycle checkpoint genes in leukemia

**Fig. 3:**
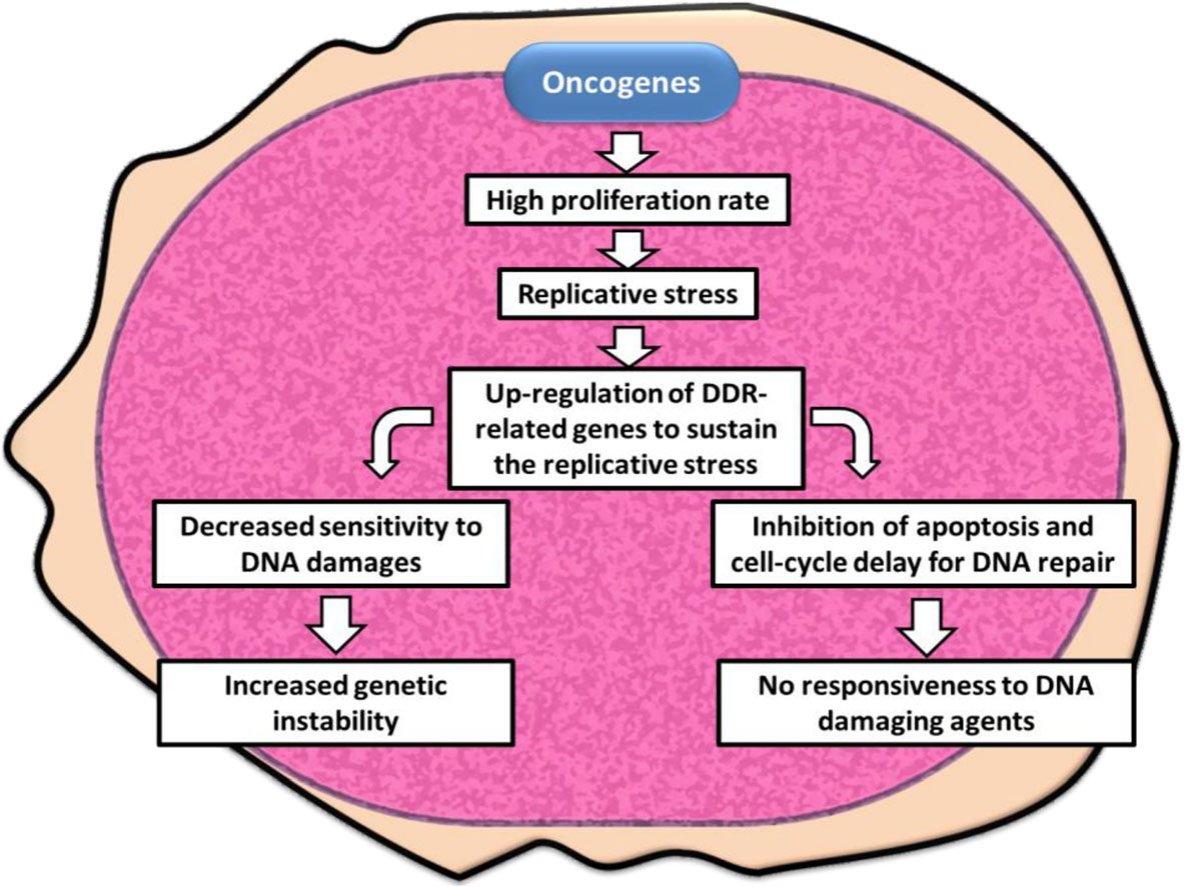
The DDR pathway in cancer cells. The high proliferation rate induced by different oncogenes (MYC or BCR-ABL1) can led to the so called replicative stress which is a negative signal for proliferation. In order to sustain the replicative stress and continue to proliferate, leukemic cells need to up-regulate different key elements of the DDR pathway, like CHK1. The two most important consequences of DDR elements up-regulation are (1) genetic instability due to the increment of tolerable level of DNA damages and (2) resistance to DNA damaging agents, such as chemotherapies, due to the up-regulation of the mechanisms involved in the DNA repair

In highly proliferating tumor cells, the activation of different oncogenes causes the so called replicative stress and, consequently, the activation of different elements of the DDR [55, 56]. This phenomenon has been thought to participate in the early phases of tumor progression and, at least in solid tumors, with the development of pre-neoplastic lesions. In particular, the dysregulation of DDR-related genes together with the activation of specific oncogenes is responsible for the high genetic instability that characterizes acute leukemia. Different groups have reported that the activation of oncogenes, like MYC, BCR-ABL1, and FLT3/ITD, alters the expression of different genes involved in the response to DNA damages. Today is generally believed that MYC-driven cells in order to sustain the high proliferative state induced by MYC itself need to up-regulate the expression of genes involved in both ATR/CHK1 and ATM/CHK2 pathway. In particular, in MYC-driven B cell lymphomas, the hyper-activation of the ATR/CHK1 pathway is thought to be fundamental to protect the replicative forks from collapse [57, 58]. MYC has been found overexpressed not only in lymphoma cells but also in chronic myeloid leukemia (CML) patients [59], in ALL patients harboring the translocations t(8;14), t(8;22), and t(2;8) [60] and in AML [61]. In a recent study, Muvarak and colleagues showed that in BCR-ABL1 and FLT3/ITD-positive leukemia cells, the constitutive activation of these kinases, via the overexpression of MYC, triggers intracellular pathways that increase genomic instability through generation of ROS, DSBs, and error-prone repair [62]. A study from Cavelier C. and colleagues showed that in primary AML samples with complex karyotype, the level of DNA damage detected by phospho-H2AX as well as the level of activated CHK1 is higher than in AML samples with normal karyotype and in normal hematopoietic precursors [63]. In ALL, different studies have confirmed the overexpression of the kinase CHK1 in leukemic blasts in comparison with its expression in normal lymphoid precursors [64, 65]. Moreover the ATR/CHK1 pathway has been found to protect BCR-ABL1-positive leukemic cells from the cytotoxicity of conventional therapies, slowing the cell cycle progression and allowing the leukemic cells to repair the DNA damages induced by the therapeutic treatment [66] (Fig. 3).

### Cell cycle checkpoint kinase inhibitors against leukemias

Due to the central role in the DNA damage response, different cell cycle checkpoint inhibitors (ATM/ATR/CHK1/CHK2/WEE1 inhibitors) have been developed to specifically inhibit the mechanisms by which tumor cells respond to DNA damaging agents. Initially, this class of compounds has been developed for the treatment of p53 mutated tumors because of their impaired G1/S checkpoint, and then, their applicability has been extended also to p53 wild-type tumors [42, 43]. These compounds have been developed to potentiate the efficacy of different chemotherapeutic compounds especially for the treatment of solid tumors [44]. The following section summarizes the main studies that have been performed to explore the efficacy of different cell cycle checkpoint inhibitors in acute (ALL and AML) and chronic (CLL and CML) leukemias.

#### ATM/ATR inhibitors against leukemias

##### ATM inhibitors

KU-55933 was the first developed potent ATM inhibitor (KuDOS Pharmaceuticals, AstraZeneca). Hickinson and colleagues showed that KU-55933 confers marked sensitization to ionizing radiation and DNA DSB-inducing chemotherapeutics, such as the topoisomerase II inhibitors (etoposide and doxorubicin), in cancer cells [67]. The efficacy of KU-55933 against leukemic cells was evaluated in Jurkat cells with or without etoposide. The combination between the DSBs inducer and the ATM inhibitor deeply affected the cell viability of the leukemic cells [68]. Although KU-55933 showed strong efficacy in vitro, its high lipophilicity limited the use in in vivo studies.

KU-59403 is a novel ATM inhibitor with improved potency, solubility, and bioavailability over the KU-55933. Batey and colleagues demonstrated a good tissue distribution and a good efficacy in mice [69]. In acute leukemia, Grosjean-Raillard and colleagues demonstrated that treatment with KU-59403 represses the antiapoptotic transcription factor nuclear factor-κB (NF-κB) pathway, which has been found to be constitutively activated in CD34+ myeloblasts of high-risk myelodysplastic syndrome (MDS) and AML patients and consequently, it induces cell death via apoptosis [70]. Despite none clinical trials have been yet performed using ATM inhibitors, the results of several in vitro studies carried out that the pharmacological inhibition of this protein has great potential as a cancer therapy in combination with radiotherapy or certain chemotherapeutic drugs (like topoisomerase inhibitors).

##### ATR inhibitors

Schisandrin B was the first ATR-selective small molecule inhibitor that has been evaluated in vitro. Nishida and colleagues reported that schisandrin B was able to abrogate UV-induced intra-S phase and G2/M cell cycle checkpoints and increase the cytotoxicity of UV radiation in human lung cancer cells [71]. Then, Vertex Pharmaceuticals using a large high-throughput screening led to the discovery of the first series of both potent and selective ATR kinase inhibitors [72]. The first selective ATR inhibitor, VE-821, had >100-fold selectivity for ATR versus ATM, PI3K, DNA-PK, and mTOR and sensitized leukemic cell lines to radiotherapy [72, 73].

VE-822 (VX-970), a further analogs of VE-821, has been improved with increased solubility, potency, selectivity, and pharmacodynamic properties [74]. Several pre-clinical studies have shown that VX-970 robustly sensitizes multiple tumor cell lines to cisplatin, ionizing radiation, gemcitabine, PARP inhibitors, topoisomerase I inhibitors, etoposide, and oxaliplatin in vitro [75–80]. In vivo studies using both VE-821 and VX-970 showed robust results. Indeed, these two ATR inhibitors synergized with radiotherapy and gemcitabine in pancreatic cancer xenograft models [76, 77] and with irinotecan in a colorectal cancer model [79]. Nowadays, different clinical trials are ongoing against solid tumors to assess the safety, tolerability, and pharmacokinetics of VX-970 in combination with cytotoxic chemotherapy (NCT02157792, NCT02595931, NCT02567422, and NCT02595892).

AZD6738 is the second ATR inhibitor currently in clinical development that possesses significantly improved solubility bioavailability and pharmacokinetic properties compared to other ATR inhibitors and is suitable for oral dosing [81]. Treatments with AZD6738 inhibit the phosphorylation of CHK1 while increasing phosphorylation of γH2AX in vitro. In in vivo models, combinatorial studies with carboplatin or ionizing radiation (IR) demonstrated significantly reduction of tumor progression in comparison with the effects of the single treatments [81, 82]. In hematological malignances, AZD6738 showed activity as monotherapy in mantle cell lymphoma xenograft mouse models with ATM and p53 deficiencies [83] and in primary CLL patient-derived xenografts with 11q deletion (ATM deficient) and 17p deletion (p53 deficient) [84]. Finally, preliminary data highlighted that AZD6738 synergizes with carboplatin, bendamustine, and cyclophosphamide in an ATM-deficient diffuse large B cell lymphoma model. Currently, no data have been published using ATR inhibitors in acute leukemia.

#### CHK1/CHK2 inhibitors against leukemias

**Fig. 4:**
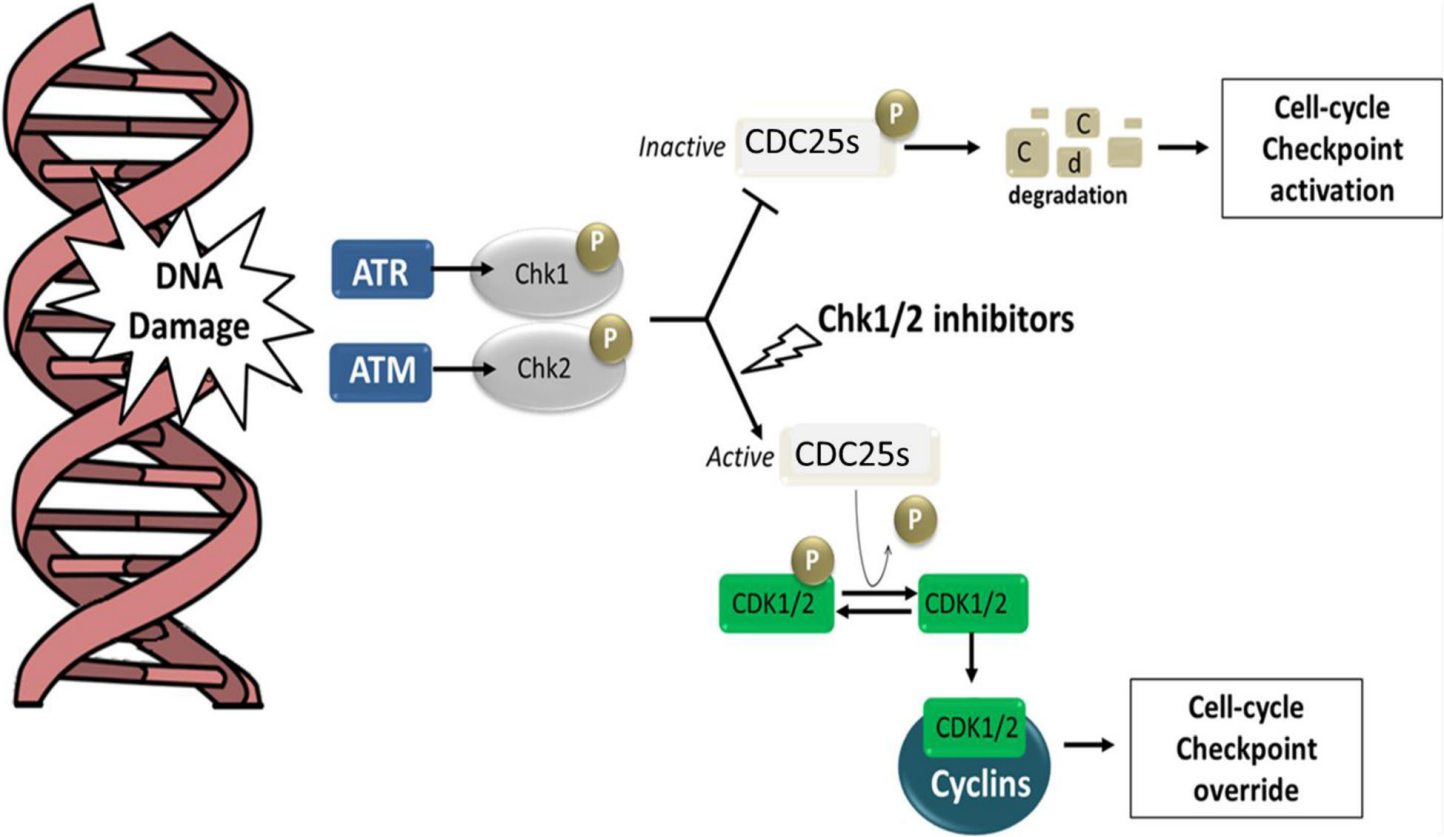
Schematic representation of the mechanism of action of CHK1/CHK2 inhibitor. In both normal and tumor cells, the recognition of damages on DNA by the DDR-sensors activates different cell cycle checkpoints. The central event of checkpoint activation is the inhibition of the phosphatases CDC25s which is necessary for the activation of the complexes CDK-cyclins. Both ATR/CHK1 and ATM/CHK2 pathways promote CDC25s inhibition (ubiquitin-dependent degradation) and, consequently, they arrest cell cycle in response to DNA damages. Tumor cells can activate these pathways in response to DNA damaging agents and survive. The treatment with a CHK1/CHK2 inhibitor avoids the degradation of the phosphatase CDC25s, inducing cell cycle progression even in the presence of DNA damages. For this reason, different CHK1/CHK2 inhibitors have been developed to enhance the DNA damaging from chemotherapeutic drugs by inhibiting the cell cycle checkpoint negative signals

In the last decade, the number of publications evaluating the pre-clinical and clinical efficacy of small molecule inhibitors of CHK1 has constantly grown [85] as well as the number of molecules against this kinase [86, 87] (Fig. 4). The first inhibitor of CHK1 was the UCN-01 (known as 7-hydroxystaurosporine). This molecule showed to inhibit not only CHK1 but also other different kinases (CHK2, CDK1, CDK2, PKC 7, and MK2) and to promote the G2/M checkpoint override upon treatment with DNA damaging agents such as cisplatin or topoisomerase inhibitor. UCN-01 was tested in several clinical trials; however, the low specificity of the compound caused many harmful side effects and this avoided its progression beyond phase II clinical trials [88, 89].

MK-8776 (SCH900776) is a potent and selective CHK1 inhibitor in clinical development. It rapidly, less than 2 h, induced γH2AX accumulation and suppressed CHK1 functionality (shown by the reduction of the auto-phosphorylation site of serine 296). The efficacy of this inhibitor was assessed not only in single agent but also in combination with different genotoxic compounds showing chemotherapy sensitization by increasing the level of DSBs. Many other studies confirmed the great efficacy of this compound for the treatment of different kinds of tumor, and today, MK-8776 entered in phase II clinical trials in combination with chemotherapy [86, 90–92]. The efficacy of the compound was also evaluated in hematological malignances. Day and colleagues demonstrated that MK-8776 synergistically potentiated the histone deacetylase (HDAC) inhibitor (HDACI) vorinostat in both AML cell lines and primary cells [92]. Moreover, they showed that efficacy of the combination was independent on the mutational status of p53 and that the synergistic interactions were associated with inhibition of CHK1 activity, interference with the intra-S phase checkpoint, disruption of DNA replication, and down-regulation of proteins involved in DNA replication and repair [92]. Zemanova J. and colleagues reported that SCH900776 enhanced the cytotoxicity of different nucleoside analogs (fludarabine, cytarabine, and gemcitabine) on the p53-deficient CLL cell line MEC1 and primary cells isolated from CLL patients [93].

AZD7762 is an ATP competitive CHK1/CHK2 inhibitor. This compound was evaluated in different trials as a chemo-sensitizer agent for conventional chemotherapy. It has been described that lung cancer cells expressing high levels of CHK1 were hyper-sensitive to AZD7762. This suggests a correlation between CHK1 inhibitor-mediated sensitivity and elevated amounts of CHK1. Different further studies were performed to investigate the efficacy of ASD7762 in combination with different compounds. Indeed, it has been reported that combination of AZD7762 with gemcitabine and ionizing radiation deeply sensitized pancreatic cells to radiation [94]. The efficacy of the compound was evaluated also in hematologic malignances, e.g., in different myeloma multiple (MM) cell lines. The combination of AZD7762 with alkylating agents (melphalan) promoted apoptosis and mitotic catastrophe of p53-mutated MM cells [95]. Moreover, Didier et al. showed that AZD7762 enhances genotoxic treatment efficacy in immature KG1 AML cell line and in AML primary leukemic cells [96]. In this study, they also found a correlation between the sensitivity to the checkpoint kinase inhibitors and a complex karyotype, usually a poor prognostic marker to conventional chemotherapy. Thus, the basal level of DNA damage (γH2AX, CHK1, and phosphorylated ATM/ATR substrates) could be a useful marker to select AML patients susceptible to receive this type of combination therapy [96].

PF-0477736 is a selective and competitive inhibitor for the CHK1 ATP site. Its specificity is 100 times stronger for CHK1 than that for CHK2. The efficacy of this compound has been well established against different kinds of tumor. In ovarian cancer, it has been shown that tumor cells strongly respond to treatment with PF-0477736 but they generate metastasis and chemo-resistant clones [97]. The efficacy of PF-0477736 has been evaluated also in leukemia. Sarmento et al. [65] demonstrated that the T-ALL primary samples express higher level of CHK1 kinase in comparison to normal thymocytes. The treatment with PF-0477736 promoted apoptotic cell death and CHK1 inhibition and consequently impaired replication and abrogation of G2/M checkpoint in T-ALL cells. Interestingly, in vitro treatment did not significantly affect the viability of normal thymocyte cells [65]. Similar results have been shown by our group. The inhibition of CHK1/CHK2 by PF-0477736 as single agent deeply reduced the cell viability of ALL primary cells and leukemia cell lines. The results from the in vitro/ex vivo studies were further confirmed using an in vivo model [64]. Recently, Nguyen T. and colleagues reported the in vitro/in vivo synergic efficacy of PF-00477736 in combination with the Src/ABL inhibitor bosutinib (SKI-606) in BCR-ABL1-positive CML or ALL cells, focusing on highly imatinib-resistant models with ABL kinase mutations. The authors speculated that the combination acts through a BCR-ABL1-independent process that may involve multiple mechanisms, including inactivation of ERK1/2 and Src, up-regulation of BIM, down-regulation of MCL-1 (BCL-2-like protein), activation of CDK1, and induction of DNA damage [98].

LY2603618, a potent and selective inhibitor of CHK1, is the first second-generation checkpoint kinase inhibitor that has been evaluated in a clinical trial [99]. King and colleagues [100] reported that the treatment with LY2603618 produced a cellular phenotype similar to that reported for depletion of CHK1 by RNA interference (RNAi). Moreover, they reported that the inhibition of CHK1 caused impaired DNA synthesis, elevated H2AX phosphorylation, and pre-mature entry into mitosis. Finally, they showed that LY2603618 was able to override the G2/M checkpoint activated after the exposure to doxorubicin, resulting in cells entering into metaphase with poorly condensed chromosomes [100]. In several studies, LY2603618 potentiated the effect of DNA damage compounds like pemetrexed and cisplatin in vitro. This result was confirmed in vivo using a tumor xenograft model and placed the bases for a phase I clinical trial evaluating the effectiveness of LY2603618 in combination with pemetrexed and cisplatin in patients with advanced cancer [99]. Zhao J. and colleagues have recently reported the efficacy of LY2603618 in combination with the BCL-2 inhibitor ABT-199 in AML cell lines and primary cells (*n* = 26). The authors demonstrated that the treatment with LY2606368 reduced the total amount of MCL-1 and, consequently, enhanced the efficacy of ABT-199 in terms of induction of apoptosis [101].

LY2606368 (prexasertib) is a novel CHK1/CHK2 inhibitor which has been reported to cause as a single agent DBSs while simultaneously removing the protection of the DNA damage checkpoints. King and colleagues reported that LY2606368 increases extensive DNA damage in the cell population in S phase highlighting the possible mechanism of death through replication catastrophe [102]. In a recent study from our group in acute lymphoblastic leukemia, the efficacy of LY2606368 was evaluated both as single agent and in combination with different compounds currently used in clinical practice. This study showed that LY2606368 deeply sensitized both primary and leukemic cells to the antimetabolite, clofarabine, and to tyrosine kinase inhibitors (imatinib and dasatinib) [103].

#### WEE1 inhibitors against leukemias

**Fig. 5:**
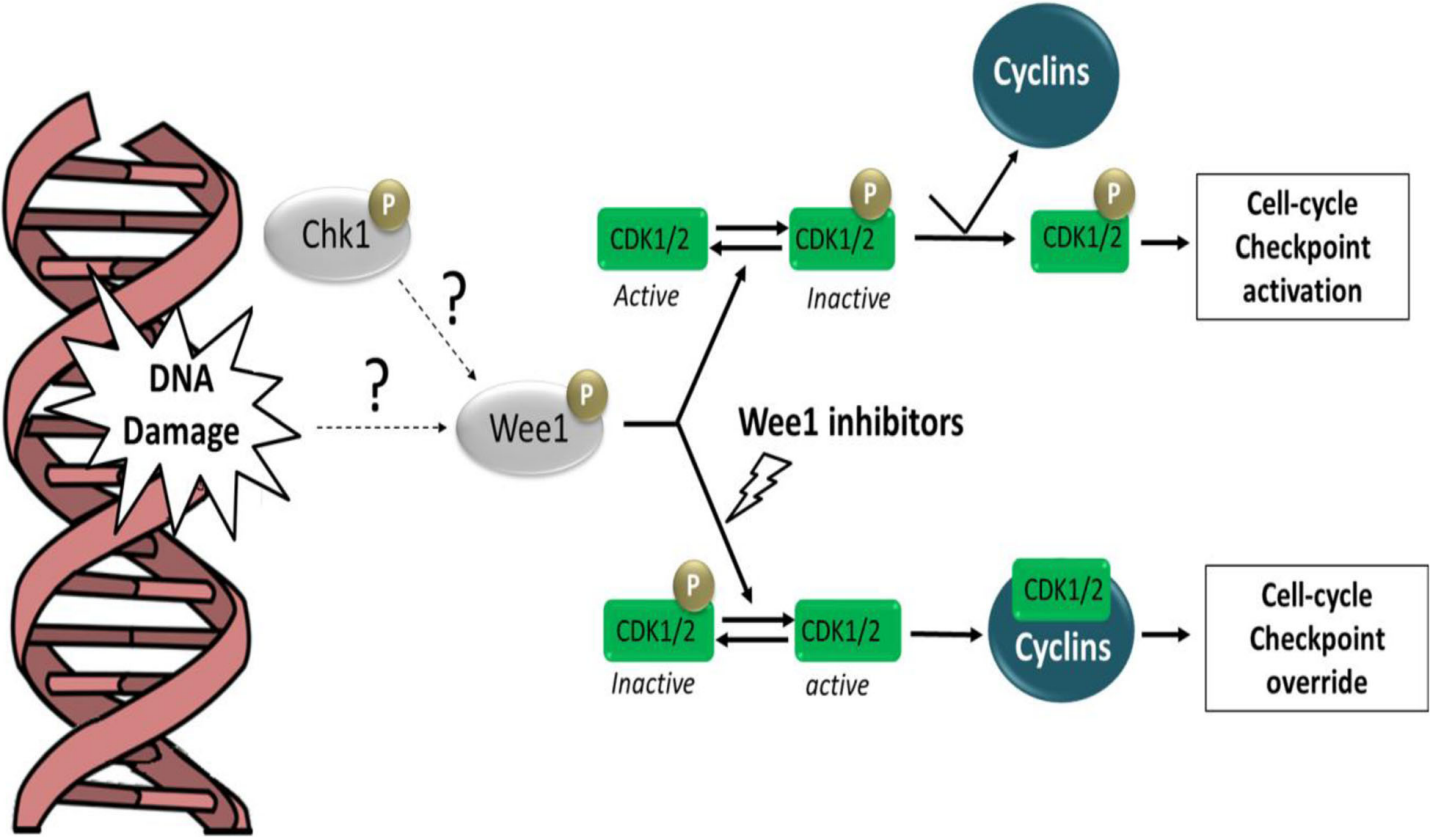
Schematic representation of the mechanism of action of the WEE1 inhibitor. In both normal and tumor cells, the activation of WEE1 upon induction of DNA damage is not fully understood. WEE1 phosphorylates both CDK1/CDK2 to prevent the constitution of the complexes CDK/cyclins. Inhibition of WEE1 activity prevents the phosphorylation of CDKs and impairs the cell cycle checkpoint after DNA damage induction. This may lead to apoptosis upon treatment with DNA damaging chemotherapeutic agents

Many WEE1 inhibitors have been developed to override cell cycle checkpoint signaling and, consequently, to improve the sensitivity of tumor cells to the toxic effect of different genotoxic agents (Fig. 5). Several studies have shown their efficacy in the treatment of different kinds of tumor not only in combinatorial studies but also as single agent. The PD0166285 is a non-selective kinase inhibitor, which targets WEE1 but also CHK1, Myt1, c-Src, PDGFR-, fibroblast growth factor receptor-1, and epidermal growth factor receptor tyrosine kinases [104, 105]. This inhibitor has been shown to successfully inhibit CDK1 phosphorylation (tyrosine 15 and threonine 14) and to abrogate G2/M checkpoint after IR irradiation in vitro.

MK-1775 (AZD1775) is the mostly studied WEE1 inhibitor. Several studies have shown that this inhibitor selectively sensitizes p53-deficient cancer cells to the toxic effect of gemcitabine, carboplatin, 5-fluorouracil, and cisplatin [106–109]. The sensitizing activity of MK-1775 selectively on p53-deficient cells has been shown also after gamma ray irradiation. Although all the above mentioned studies, recent findings highlighted that the effectiveness of this compound in different types of tumor is independent on the functional status of p53 [110]. In hematological malignancies, recent studies mainly on acute myeloid leukemia have shown the efficacy of this compound, not only as single agent [111] but also in combination with different compounds like HDAC (vorinostat), CDK inhibitor (roscovitine), LY2603618, or cytarabine [111–116]. The combination of cytarabine and MK-1775 enhanced chemotherapy cytotoxicity by abrogating the mechanisms of DNA repair and by the inhibition of the S phase arrest induced by cytarabine [110, 117]. Similar results were recently found in T-ALL cell lines and in in vivo models [118]. Tibes and colleagues showed the efficacy of MK-1775 in both CML primary cell and cell lines as single agent and in combination with cytarabine. In this study, they showed that the inhibition of WEE1 significantly sensitizes leukemic cells to cytarabine in terms of reduction of cell viability and induction of apoptosis [117]. These data support the development of clinical trials including AZD1775 in combination with conventional chemotherapeutic compounds for leukemias.

### Data available from clinical trials in solid tumors

A phase I dose-escalation study aiming to examine the safety and tolerability of LY2603618 in combination with pemetrexed 500 mg/m^2^ every 21 days in patients with cancer defined a maximum tolerated dose (MTD) of 150 mg/m^2^. A following phase I study using LY2603618 in combination with gemcitabine in patients with solid tumors showed that among the 50 patients enrolled, frequent adverse events, possibly related to study drug treatment, included fatigue (44%), decreased platelets (42%), decreased neutrophils (32%), nausea (26%), and decreased hemoglobin (20%). Systemic exposure of LY2603618 increased dose dependently, while clearance was relatively dose independent. The mean LY2603618 half-life varied. However, the durations were still suitable for maintaining human exposures while minimizing accumulation. LY2603618 pharmacokinetic (PK) was not altered by gemcitabine administration. Plasma exposures that correlate with the maximal pharmacodynamic effect in non-clinical models were achieved for all doses. One patient with non-small cell lung cancer carcinoma achieved a partial response; 22 patients had stable disease. They conclude that the MTD of LY2603618 combined with gemcitabine was 200 mg/m^2^, but a fixed LY2603618 dose of 230 mg combined with gemcitabine was selected as the recommended phase II dose [99]. A consequent phase II study evaluating the effect of LY2603618 in combination with pemetrexed in patients with advanced or metastatic non-small cell lung cancer highlighted no significant improvement of pemetrexed efficacy as single agent in non-small cell lung cancer [119]. Until now, only a phase I study has been done using LY2606368 as single agent in advanced solid tumors. Forty-five patients were treated with two different dose-escalation schedule: from 10 to 50 mg/m^2^ on schedule 1 (days 1 to 3 every 14 days) or from 40 to 130 mg/m^2^ on schedule 2 (day 1 every 14 days); seven experienced dose-limiting toxicities (all hematologic). The MTDs were 40 mg/m^2^ (schedule 1) and 105 mg/m^2^ (schedule 2). The most common related grade 3 or 4 treatment-emergent adverse events were neutropenia, leukopenia, anemia, thrombocytopenia, and fatigue. Grade 4 neutropenia occurred in 73.3% of patients and it was transient (typically <5 days). Febrile neutropenia incidence was low (7%). The LY2606368 exposure over the first 72 h (area under the curve from 0 to 72 h) at the MTD for each schedule coincided with the exposure in mouse xenografts that resulted in maximal tumor responses. Minor intra- and intercycle accumulation of LY2606368 was observed at the MTDs for both schedules. Two patients (4.4%) had a partial response. Fifteen patients (33.3%) had a best overall response of stable disease (range, 1.2 to 6.7 months), six of whom had squamous cell carcinoma. An LY2606368 dose of 105 mg/m^2^ once every 14 days is being evaluated as the recommended phase II dose in dose-expansion cohorts for patients with squamous cell carcinoma. A phase I study of single-agent AZD-1775 involving 25 patients with refractory solid tumors showed that the MTD was established as 225 mg twice per day orally over 2.5 days per week for 2 weeks per 21-day cycle. Confirmed partial responses were observed in two patients carrying *BRCA* mutations: one with head and neck cancer and one with ovarian cancer. Common toxicities were myelosuppression and diarrhea. The on-target efficacy of the compound was assessed looking at the levels of phosphorylated Tyr15-Cdk (pY15-Cdk) and γH2AX in paired tumor biopsies obtained at the MTD [120]. A second phase I study demonstrated target inhibition (Tyr15-Cdk) at MTD in combination with carboplatin adult patients with advanced solid tumors (NCT00648648). Patients with p53 mutated ovarian cancer refractory or resistant (<3 months) to standard first line therapy (carboplatin plus paclitaxel) were re-exposed to carboplatin (AUC 5), plus five bi-daily doses of 225 mg AZD-1775 in a 21-day cycle (MTD). Bone marrow toxicity, fatigue, diarrhea, nausea, and vomiting were the most common adverse events. Out of 24 patients enrolled, 22 patients were evaluable for study endpoints. As best response (RECIST 1.0), six patients (27%) showed confirmed partial response (PR) with a median progression-free survival (PFS) of 10.9 months. Nine patients (41%) had stable disease and seven patients (32%) had progressive disease as best response, with a median PFS of 5.3 and 1.3 months, respectively (NCT01164995).

## Conclusions

Nowadays, the amount of pre-clinical data has confirmed the efficacy of different cell cycle checkpoint inhibitors against different kinds of hematologic as well as solid tumors, as single agent, or in combination with a wide number of drugs. The efficacy as well as the safety of different combinations is now being established also in several phase I/II clinical trials. Most of the studies were based on the use of cell cycle checkpoint inhibitors in combination with standard chemotherapy in order to enhance its effectiveness. Although the good successes that have been achieved have many questions needed to be answered regarding the safety and the effectiveness of this class of compounds. Some acute leukemia subtypes are characterized by high genetic instability that should make this kind of tumor very sensitive to cell cycle checkpoint inhibitors. However, few clones can take advantage from the inhibition of DNA repair, acquire novel invasive features, and start to proliferate. Long-period safety of cell cycle checkpoint inhibitors should be addressed also in normal tissues in order to exclude tumor transformation of healthy cells. A second crucial question that should be answered, at least in hematological malignances, is the ability of cell cycle checkpoint inhibitors to eradicate leukemic stem cells in the contest of bone marrow niche. Indeed, until today, very few studies have addressed, for example, the efficacy of the checkpoint inhibitors under hypoxic condition or more general in experimental settings that mime the niche micro-environment. Finally, prognostic markers should be evaluated to stratify patients that could be more sensitive to checkpoint kinase inhibitors. One predictive marker could be the evaluation of basal expression of elements involved in the DDR and the level of genetic instability (γH2AX expression). In our opinion, based on the results from the clinical trials, a last important question should be answered: can we substitute DNA damaging agents (chemotherapy) with DDR inhibitors in standard therapeutic regimens in which a specific inhibitor, for example, BCR-ABL1 inhibitors, is associated with conventional chemotherapy? Several studies have been done to evaluate the chemotherapy-induced genetic instability in various types of cancers [121–123]. It is generally believed that DNA damaging compounds can positively select tumor cells that harbor particular mutations or can increase genetic instability leading to the generation of novel clones with more aggressive phenotypes. These two scenarios are the biological explanations for the failure of standard chemotherapy and for tumor relapses. For the abovementioned reasons, we speculate that a winning strategy to avoid relapse may be to substitute chemotherapy with cell cycle checkpoint inhibitors in the treatment of hematological malignances that can be treated with specific targeted inhibitors.

## Abbreviations

ALL: Acute lymphoblastic leukemia
AML: Acute myeloid leukemia
ATM: Ataxia-telangiectasia mutated
ATR: ATM and Rad3 related
ATRIP: ATR-interacting protein
CDC25: Cell division cycle 25
CDK: Cyclin-dependent kinases
CDKI: CDK inhibitors
CDKN1A: Cyclin-dependent kinase inhibitor 1A
CHK1: Checkpoint kinase 1
CHK2: Checkpoint kinase 2
CLL: Chronic lymphocytic leukemia
CML: Chronic myeloid leukemia
DDR: DNA damage response
DNA-PK: DNA-dependent protein kinase
MCM: Mini-chromosome maintenance
MRN: Mre11-Rad50-Nbs1
MYT1: Membrane-associated tyrosine/threonine 1
ROS: Reactive oxygen species
RPA: Replication protein A
SSB: Single-strand break
ssDNA: Single-strand DNA
TopBP1: Topoisomerase II binding protein 1
